# Efficacy and safety of subcutaneous mosunetuzumab in combination with lenalidomide and as a monotherapy in Japanese patients with relapsed/refractory follicular lymphoma

**DOI:** 10.1007/s10147-025-02957-1

**Published:** 2026-01-12

**Authors:** Shinichi Makita, Koji Izutsu, Yuko Mishima, Takahiro Kumode, Junya Kuroda, Nobuhiro Kanemura, Noriko Fukuhara, Kazuyuki Shimada, Chiemi Mori, Atsuko Kawasaki, Takeshi Miyake, Dai Maruyama

**Affiliations:** 1 Department of Hematology, National Cancer Center Hospital, Tokyo, Japan; 2 Department of Hematology Oncology, Cancer Institute Hospital, Japanese Foundation for Cancer Research, Tokyo, Japan; 3 Department of Hematology and Rheumatology, Faculty of Medicine, Kindai University, Osaka, Japan; 4 Division of Hematology and Oncology, Department of Medicine, Kyoto Prefectural University of Medicine, Kyoto, Japan; 5 Department of Hematology and Infectious Disease, Gifu University Hospital, Gifu, Japan; 6 Department of Hematology, Tohoku University, Sendai, Japan; 7 Department of Hematology and Oncology, Nagoya University Graduate School of Medicine, Nagoya, Japan; 8 Chugai Pharmaceutical Co., Ltd, Tokyo, Japan

**Keywords:** Mosunetuzumab, Lenalidomide, Follicular lymphoma, Bispecific antibodies, Subcutaneous, Japan

## Abstract

**Background:**

JO40295 (jRCT2080223801) evaluated the efficacy and safety of subcutaneous (SC) mosunetuzumab, in combination with lenalidomide and as monotherapy, in Japanese patients with relapsed/refractory (R/R) follicular lymphoma (FL). We report outcomes from the interim analysis of the FLMOON-2 (≥ 1 prior therapy; mosunetuzumab plus lenalidomide) and primary analysis of the FLMOON-3 (≥ 2 prior therapies; mosunetuzumab monotherapy) cohorts.

**Methods:**

Mosunetuzumab SC was administered with Cycle (C)1 step-up dosing in both cohorts: C1 Day (D)1, 5 mg; C1D8, D15 and C2 onwards, 45 mg. In FLMOON-2, oral lenalidomide was administered from C2 onwards, on D1–21 of each cycle. Treatment was administered up to C12 in FLMOON-2 and C8 or C17 in FLMOON-3. The primary endpoint was independent review facility-assessed complete response (CR) rate.

**Results:**

At the clinical cut-off date (FLMOON-2: April 4, 2024; FLMOON-3: March 4, 2024), in the efficacy-evaluable populations, CR rate was 92.3% in FLMOON-2 (n = 13) and 100% in FLMOON-3 (n = 5). In the safety-evaluable populations (FLMOON-2, n = 17; FLMOON-3, n = 5), Grade 3/4 adverse events (AEs) occurred in 64.7% of patients in FLMOON-2 and 20.0% in FLMOON-3. No Grade 5 AEs or AEs leading to treatment discontinuation occurred in either cohort. Cytokine release syndrome was reported in 47.1% of patients in FLMOON-2 and 20.0% in FLMOON-3. Serum mosunetuzumab concentration peaked with the third dose of mosunetuzumab in C1 and reached a steady state with repeated dosing.

**Conclusion:**

Mosunetuzumab SC, in combination with lenalidomide and as monotherapy, demonstrated promising efficacy with a manageable safety profile in Japanese patients with R/R FL.

**Supplementary Information:**

The online version contains supplementary material available at 10.1007/s10147-025-02957-1.

## Introduction

Follicular lymphoma (FL) is the second most common non-Hodgkin lymphoma (NHL) subtype in the United States (US) and Western Europe, and also in Japan where it represents approximately 20% of all cases [1, 2]. Despite advances in treatment approaches for FL, many patients experience disease relapse, and survival outcomes decline with each subsequent line of therapy [3]. Until recently, treatment options for patients with relapsed/refractory (R/R) FL in Japan were limited to rituximab, with or without chemotherapy, and chimeric antigen receptor T-cell therapy [4]. However, the emergence of bispecific antibody therapy has changed the treatment landscape [5].

Mosunetuzumab is an off-the-shelf CD20xCD3 T-cell engaging bispecific antibody that redirects T cells to eliminate B cells, including those that cause malignant disease, and was the first bispecific antibody granted regulatory approval for R/R FL, in Europe and the US in 2022 [6, 7]. In a global pivotal Phase I/II study (GO29781; NCT02500407), intravenous (IV) mosunetuzumab induced high and durable complete responses (CRs), and had a manageable safety profile in patients with R/R FL after ≥ 2 prior lines of therapy [8, 9]. Mosunetuzumab IV has also been evaluated in Japanese patients with R/R B-cell NHL after ≥ 2 prior lines of therapy, and was approved for the treatment of R/R FL after ≥ 2 prior lines of therapy in Japan in 2024 [10, 11]. Mosunetuzumab IV in combination with lenalidomide is currently being investigated in a Phase Ib/II study (CO41942; NCT04246086) in patients with R/R FL after ≥ 1 prior line of therapy. Initial results demonstrated that mosunetuzumab in combination with lenalidomide had a manageable safety profile and encouraging preliminary anti-tumor activity [12]. This combination is also being studied in the global Phase III CELESTIMO trial (NCT04712097) in patients with R/R FL after ≥ 1 prior line of therapy [13].

A subcutaneous (SC) formulation of mosunetuzumab has been developed to facilitate more convenient delivery compared with IV administration and was approved in Europe in 2025, for the treatment of patients with R/R FL after ≥ 2 prior lines of therapy [14, 15]. In a Phase I/II study of patients with R/R FL, the pharmacokinetics of mosunetuzumab SC were shown to be non-inferior versus the IV formulation; mosunetuzumab SC achieved deep and durable responses, with a manageable safety profile and low incidence and severity of cytokine release syndrome (CRS) [14]. These findings suggest that the SC formulation could further enhance the benefits of mosunetuzumab with improved patient convenience and practice efficacy [14].

Here, we present results from JO40295 (jRCT2080223801), the first to evaluate mosunetuzumab SC in combination with lenalidomide in patients with R/R FL and ≥ 1 prior line of therapy (FLMOON-2 cohort; interim analysis) and as a monotherapy in patients with ≥ 2 prior lines of therapy (FLMOON-3 cohort; primary analysis), in the Asia–Pacific region.

## Materials and methods

### Study design

This Phase I, Japanese multicenter, open-label study was designed to evaluate the safety, tolerability, pharmacokinetics, anti-tumor activity and immunogenicity of mosunetuzumab in combination with lenalidomide and as a monotherapy in patients with R/R B-cell NHL.

Eligible patients were aged ≥ 18 years with histologically confirmed CD20 + Grade 1–3a FL and an Eastern Cooperative Oncology Group performance status of 0–2. Patients from the FLMOON-2 cohort had received ≥ 1 prior regimens of systemic lymphoma treatment (including immunotherapy or chemoimmunotherapy). In the FLMOON-3 cohort, patients had received ≥ 2 prior regimens of systemic lymphoma therapy, including an anti-CD20 targeted therapy and an alkylating agent. Full inclusion and exclusion criteria can be found in Online Resource.

The protocol was approved by the relevant institutional review boards. The trial was conducted in accordance with the Declaration of Helsinki, the International Conference on Harmonization guidelines for Good Clinical Practice, and applicable laws and regulations. All patients provided written informed consent.

### Study treatment

In both cohorts, mosunetuzumab SC was administered at the previously identified recommended Phase 2 dose with step-up dosing in Cycle 1: Cycle 1 Day 1, 5 mg; Cycle 1 Day 8, 45 mg; Cycle 1 Day 15, 45 mg; Cycle 2 Day 1 onwards, 45 mg [16]. In the FLMOON-2 cohort, Cycle 1 was a 21-day cycle and Cycles 2–12 were 28 days; in the FLMOON-3 cohort, all cycles were 21 days.

In the FLMOON-2 cohort, lenalidomide (20 mg orally) was administered from Cycle 2 onwards on Day 1–21 of each cycle. Mosunetuzumab and lenalidomide were administered up to Cycle 12, or until unacceptable toxicity or progressive disease (PD).

In the FLMOON-3 cohort, mosunetuzumab was administered for a fixed duration up to Cycle 8 or Cycle 17, or until unacceptable toxicity or PD. Patients with a CR at the end of Cycle 8 discontinued treatment. Those with stable disease or a partial response at Cycle 8 could continue treatment to Cycle 17.

### Objectives and outcome assessments

The primary objective of each cohort was to evaluate the anti-tumor activity of mosunetuzumab in combination with lenalidomide, and as a monotherapy, in patients with R/R FL who had received ≥ 1 and ≥ 2 prior therapies, respectively. The primary endpoint was CR rate as assessed by an independent review facility (IRF) using Lugano 2014 criteria [17]. Secondary endpoints included: CR rate by investigator assessment; overall response rate (ORR), duration of response (DOR), duration of complete response (DOCR) and progression-free survival (PFS) by IRF and investigator assessment; overall survival (OS); safety and pharmacokinetics.

In the FLMOON-2 cohort, tumor assessments were performed at screening, at the end of Cycles 3, 6, 9 and 12, and then every 6 months during follow-up. In the FLMOON-3 cohort, tumor assessments were performed at screening, every 3 months until treatment completion or discontinuation, every 3 months for the first 24 months during follow-up, and every 12 months thereafter. Responses were assessed by the investigator or sub-investigator and the IRF, using contrast enhanced computed tomography or fluorodeoxyglucose positron emission tomography scans.

Adverse events (AEs) were assessed using the National Cancer Institute Common Terminology Criteria for Adverse Events v.4.03 [18]. CRS was assessed using the Lee 2014 CRS grading criteria [19] and the American Society for Transplantation and Cellular Therapy (ASTCT) consensus grading criteria [20].

The key pharmacokinetic endpoint was mosunetuzumab serum concentration; pharmacokinetic parameters included maximum concentration (C_max_), time to peak drug concentration (T_max_) and trough serum concentrations.

### Statistical analyses

For the FLMOON-2 cohort, the prescribed threshold CR rate was assumed to be 30% based on the CR rate of 34% (95% confidence interval [CI]: 27–41), observed in the Phase III AUGMENT study (NCT01938001) of rituximab plus lenalidomide in patients with R/R indolent NHL [21]. The expected CR rate was conservatively set at 60.0% for the FLMOON-2 cohort, informed by the interim data from a Phase Ib/II study (CO41942) of mosunetuzumab plus lenalidomide in patients with R/R FL (clinical cut-off date [CCOD]: September 13, 2021) [12], which demonstrated a CR rate of 65.5% (95% CI: 45.7–82.1). One interim analysis was planned for this cohort.

Under these assumptions, a target sample size of 17 patients was required to have at least 80% power to detect this hypothesis; the O’Brien-Fleming type α-spending function was used to control the type 1 error probability at a one-sided significance level of 5%.

Following discussions with regulatory authorities, a target sample size of five patients was set for the FLMOON-3 cohort ahead of patient enrollment; statistical considerations were not used to determine this sample size.

Descriptive statistics were used to summarize demographics and baseline characteristics. The protocol specified that the Clopper–Pearson method would be used to calculate the 95% CI of the CR rate. However, since the planned interim analysis of the FLMOON-2 cohort used multivariate normal distribution to adjust the significance level considering the multiplicity of testing, Wald 95% CIs were also calculated for this cohort, which assume a normal distribution.

Median time-to-event endpoints were estimated using the Kaplan–Meier method and the 95% CIs of the medians were calculated with the Brookmeyer–Crowley method. Three-month event-free rates were estimated; 95% CIs were calculated using the Greenwood formula.

In the FLMOON-2 cohort, the interim analysis was conducted once 13 patients had completed the tumor assessment at the end of Cycle 6 or discontinued treatment. Therefore, the efficacy-evaluable population was defined as patients who were included in the interim analysis set. In the FLMOON-3 cohort, the efficacy analysis was conducted after all patients who remained on treatment completed the tumor assessment at 6 months; the efficacy analysis-population was defined as all patients enrolled in the study. The safety population was defined as all patients who received at least one dose of study treatment and were included in the primary analysis for safety variables.

## Results

### Patients

As of the CCOD, 17 patients were enrolled to the FLMOON-2 cohort (CCOD: April 4, 2024) and five patients to the FLMOON-3 cohort (CCOD: March 4, 2024); 16 patients in the FLMOON-2 cohort were ongoing on the study and one patient had discontinued (due to PD). All five patients in the FLMOON-3 cohort remained on study.

In the FLMOON-2 cohort, median follow-up time was 5.9 months (range: 4.4–8.1), median age was 70.0 years (range: 48–78) and most patients had received one prior line of therapy (n = 15; 88.2%; Table 1). Median mosunetuzumab treatment duration was 5.3 months (range: 2.8–7.4) and the median number of mosunetuzumab cycles received was 7 (range: 4–9); median mosunetuzumab dose intensity was 99.3% (range: 84.8–100.6). The median duration of lenalidomide treatment was 5.3 months (range: 2.8–7.2), and the median number of lenalidomide treatment cycles received was 6 (range: 3–8); median lenalidomide dose intensity was 93.7% (range: 56.9–100.6).

**Table 1:** Demographics and baseline characteristics

n (%) unless stated	FLMOON-2 Mosunetuzumab + lenalidomide (n = 17)	FLMOON-3 Mosunetuzumab monotherapy (n = 5)
Median age, years (range)	70.0 (48–78)	72.0 (43–83)
Male	9 (52.9)	1 (20.0)
Median BMI (kg/m^2^)	24.1 (18.9–31.5)	24.4 (19.8–27.7)
ECOG PS		
0	15 (88.2)	5 (100.0)
1	2 (11.8)	0
2	0	0
Ann Arbor		
Stage I	0	0
Stage II	2 (11.8)	1 (20.0)
Stage III	3 (17.6)	2 (40.0)
Stage IV	12 (70.6)	2 (40.0)
Number of prior lines of therapy		
Median (range)	1 (1–2)	2 (2–4)
1	15 (88.2)	0
2	2 (11.8)	3 (60.0)
3	0	1 (20.0)
4	0	1 (20.0)
Refractory to prior anti-CD20 therapy		
Yes	2 (11.8)	2 (40.0)
No	14 (82.4)	3 (60.0)
Unknown	1 (5.9)	0
Double-refractory to prior anti-CD20 therapy and prior alkylator therapy		
Yes	1 (5.9)	2 (40.0)
FLIPI score		
1	4 (23.5)	1 (20.0)
2	3 (17.6)	1 (20.0)
3	9 (52.9)	2 (40.0)
4	1 (5.9)	1 (20.0)
POD24		
Yes	6 (35.3)	1 (20.0)
Bulky disease > 6 cm		
Yes	11 (64.7)	3 (60.0)

*BMI* body mass index, *ECOG PS* Eastern Cooperative Oncology Group performance score, *FLIPI* Follicular Lymphoma International Prognostic Index, *POD24* progression of disease within 24 months of first-line therapy

In the FLMOON-3 cohort, median follow-up time was 6.4 months (range: 5.7–8.5), median age was 72.0 years (range: 43–83) and 3/5 patients had received two prior lines of therapy (60.0%; Table 1). Median treatment duration was 4.9 months (range: 4.9–7.2) and the median number of mosunetuzumab cycles received was 8.0 (range: 8–8); median dose intensity was 98.7% (range: 67.6–100).

### Efficacy

**Fig. 1:**
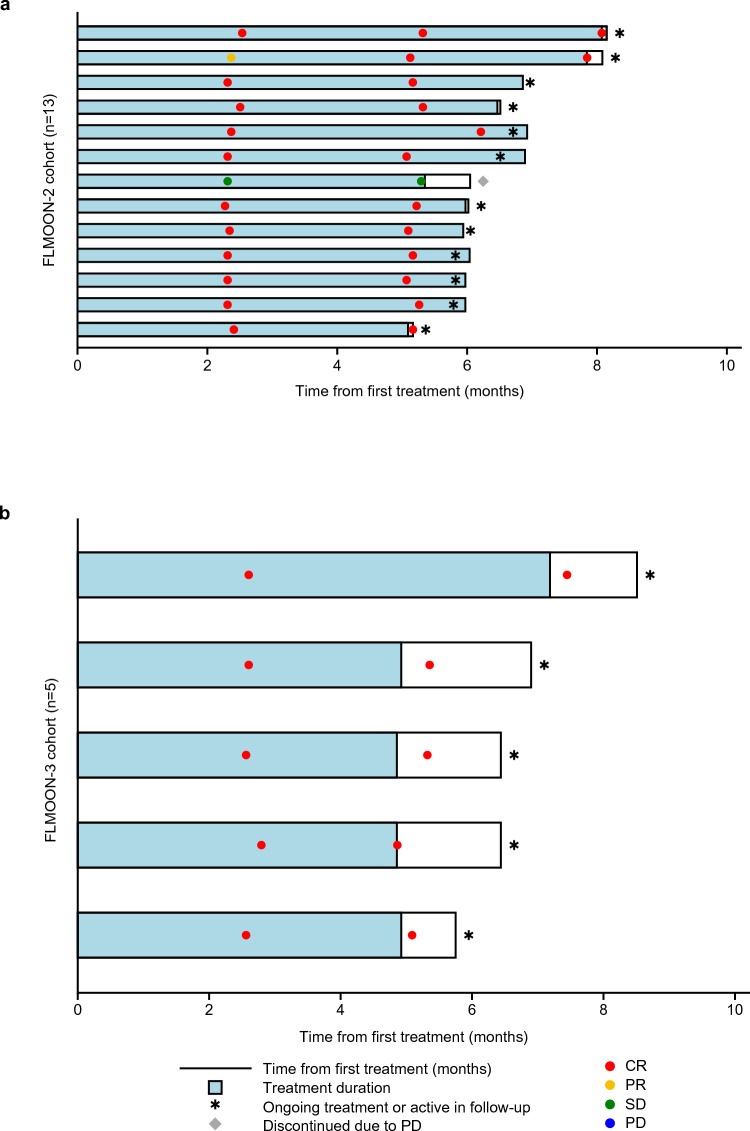
Time from first treatment swimmer plots and response by IRF assessment in the **a** FLMOON-2 (mosunetuzumab + lenalidomide; n = 13) and **b** FLMOON-3 (mosunetuzumab monotherapy; n = 5) cohorts. *CR* complete response, *IRF* independent review facility, *PD* progressive disease, *PR* partial response, *SD* stable disease

In the efficacy-evaluable population of the FLMOON-2 cohort (n = 13), the primary endpoint was achieved, and the lower limit of the 95% CI of the CR rate was higher than the prescribed threshold CR rate of 30%. The IRF-assessed CR rate in patients treated with mosunetuzumab plus lenalidomide, was 92.3% (95% CI [Wald]: 77.8–100; [Clopper–Pearson]: 64.0–99.8) and ORR was 92.3% (95% CI: 64.0–99.8) (Table 2). The median time to first CR was 2.3 months (range: 2.3–5.1; Fig. 1a). Median DOR and DOCR were not reached (NR; 95% CI: not estimable [NE]–NE) and the event-free rate at 3 months was 100% (95% CI: 100–100) for both DOR and DOCR. Median PFS was NR (95% CI: 5.3–NE); the event-free rate at 3 months was 100% (95% CI: 100–100). Median OS was NR (95% CI: NE–NE) and the event-free rate at 3 months was 100% (95% CI: 100–100).

**Table 2:** Efficacy summary

	FLMOON-2 Mosunetuzumab + lenalidomide (n = 13)		FLMOON-3 Mosunetuzumab monotherapy (n = 5)	
	IRF-assessed	Investigator-assessed	IRF-assessed	Investigator-assessed
ORR, n (%) [95% CI]	12 (92.3) [64.0–99.8]^a,b^	13 (100) [75.3–100]^a,b^	5 (100) [47.8–100]^a^	5 (100) [47.8–100]^a^
CR, n (%) [95% CI]	12 (92.3) [77.8–100]^c,d^	12 (92.3) [77.8–100]^c,d^	5 (100) [47.8–100]^a^	5 (100) [47.8–100]^a^
PR, n (%) [95% CI]	0 [0.0–24.7]^a,e^	1 (7.7) [0.2–36.0]^a,e^	0 [0.0–52.2]^a^	0 [0.0–52.2]^a^
Median DOR, months (95% CI)^f^	NR (NE–NE)	NR (NE–NE)	NR (NE–NE)	NR (NE–NE)
Median DOCR, months (95% CI)^f^	NR (NE–NE)	NR (NE–NE)	NR (NE–NE)	NR (NE–NE)
Median PFS, months (95% CI)^f^	NR (5.4–NE)	NR (NE–NE)	NR (NE–NE)	NR (NE–NE)
Median OS, months (95% CI)^f^	NR (NE–NE)		NR (NE–NE)	

*CI* confidence interval, *CR* complete response, *DOCR* duration of complete response, *DOR* duration of response, *IRF* independent review facility, *NE* not estimable, *NR* not reached, *ORR* overall response rate, *OS* overall survival, *PFS* progression-free survival, *PR* partial response

^a^Clopper–Pearson 95% CI

^b^95% CI for ORR using the Wald method was 77.8–100 for IRF assessment and 100–100 for investigator assessment

^c^Wald 95% CI

^d^95% CI for CR rate using the Clopper–Pearson method was 64.0–99.8 for IRF assessment and 64.0–99.8 for investigator assessment

^e^95% CI for PR rate using the Wald method was 0.0–0.0 for IRF assessment and 0.0–22.2 by investigator assessment

^f^95% CIs for DOR, DOCR, PFS and OS were calculated using the Brookmeyer–Crowley method

In the efficacy-evaluable population of the FLMOON-3 cohort (n = 5), the IRF-assessed CR rate was 100% (95% CI: 47.8–100.0; Table 2). The median time to first CR was 2.6 months (range: 2.6–2.8; Fig. 1b). Median DOCR was NR (95% CI: NE–NE); the DOCR event-free rate at 3 months was 100% (95% CI: 100–100). Median PFS was NR (95% CI: NE–NE) and the event-free rate at 3 months was 100% (95% CI: 100–100). Median OS was NR (95% CI: NE–NE); the event-free rate at 3 months was 100% (95% CI: 100–100).

Similar response rates were observed by investigator-assessment in both cohorts (Table 2).

### Safety

**Fig. 2:**
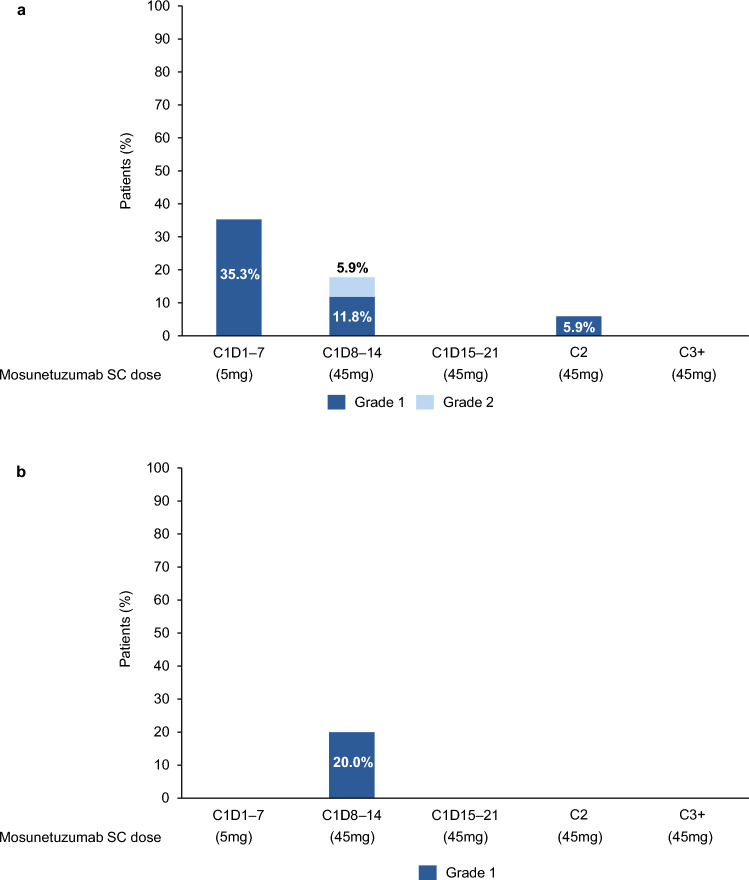
CRS by Cycle and Grade in patients from the **a** FLMOON-2 (n = 17) and **b** FLMOON-3 (n = 5) cohorts. *C* cycle, *CRS* cytokine release syndrome, *D* Day, *SC* subcutaneous

In the FLMOON-2 cohort, all safety-evaluable patients (n = 17) experienced ≥ 1 AE (Table 3). The most common AEs were injection-site reactions (ISR), which were all Grade 1 in severity and experienced by 15 (88.2%) patients. No patients discontinued treatment due to an AE, and 15 (88.2%) patients experienced ≥ 1 AE that led to dose modification/interruption. Serious AEs occurred in five (29.4%) patients, including rash in two (11.8%) patients and viral pneumonia, pyrexia, CRS, febrile neutropenia and drug-induced liver injury in one (5.9%) patient each. Of the 11 (64.7%) patients who experienced Grade 3/4 AEs (Table 3), the most common was neutropenia (n = 8; 47.1%; Table 4). No immune cell-associated neurotoxicity syndrome events occurred. Eight (47.1%) patients experienced CRS, seven (41.2%) patients had a Grade 1 event, and one (5.9%) patient had a Grade 2 event (Table 5). One patient experienced a serious CRS AE that led to a lenalidomide dose interruption. Four (23.5%) patients required treatment for the management of CRS; two (11.8%) required tocilizumab, one (5.9%) required steroids, and one (5.9%) required both steroids and tocilizumab (Table 5). Out of the ten CRS events reported, six (60.0%) occurred between Cycle 1 Day 1 and Day 7 (all Grade 1), three (30.0%) occurred between Cycle 1 Day 8 and Day 14 (n = 2, Grade 1; n = 1 Grade 2), and one (10.0%) occurred in Cycle 2 (Grade 1; Fig. 2a). No Grade 5 (fatal) AEs occurred.

**Table 3:** Safety summary

n (%)	FLMOON-2 Mosunetuzumab + lenalidomide (n = 17)	FLMOON-3 Mosunetuzumab monotherapy (n = 5)
All grade AE	17 (100)	5 (100)
Grade 3/4 AE	11 (64.7)	1 (20.0)
Grade 5 AE	0	0
AE leading to treatment withdrawal	0	0
AE leading to dose modification/interruption	15 (88.2)	1 (20.0)
Serious AE	5 (29.4)	1 (20.0)
Serious AE leading to treatment withdrawal	0	0
Serious AE leading to dose modification/interruption	4 (23.5)	1 (20.0)
Any treatment-related serious AE	4 (23.5)	1 (20.0)

*AE* adverse event

**Table 4:** Selected AEs by grade

n (%)	FLMOON-2 Mosunetuzumab + lenalidomide (n = 17)		FLMOON-3 Mosunetuzumab monotherapy (n = 5)	
	Any grade	Grade 3/4	Any grade	Grade 3/4
Cytopenia				
Neutropenia^a^	12 (70.6)	8 (47.1)	0	0
Thrombocytopenia	1 (5.9)	0	1 (20.0)	0
Febrile neutropenia	1 (5.9)	1 (5.9)	0	0
Anemia	1 (5.9)	0	0	0
Infections				
Nasopharyngeal pain	3 (17.6)	0	0	0
Pneumonia	2 (11.8)	0	0	0
Viral pneumonia	1 (5.9)	1 (5.9)	1 (20.0)	1 (20.0)
Coronavirus infection	0	0	1 (20.0)	0
Otitis media	0	0	1 (20.0)	0
Sinusitis	1 (5.9)	0	1 (20.0)	0
Cheilitis	1 (5.9)	0	0	0
Oral herpes	1 (5.9)	0	0	0
Tinea pedis	1 (5.9)	0	0	0
Neurological disorders				
Insomnia	1 (5.9)	0	2 (40.0)	0
Presyncope	1 (5.9)	0	0	0
Headache	1 (5.9)	0	0	0
Cytokine release syndrome	8 (47.1)	0	1 (20.0)	0

*AE* adverse event

^a^Grouped term includes patients who experienced neutrophil count decrease and neutropenia

**Table 5:** CRS summary

n (%), unless stated	FLMOON-2 Mosunetuzumab + lenalidomide (n = 17)	FLMOON-3 Mosunetuzumab monotherapy (n = 5)
CRS (any grade)	8 (47.1)	1 (20.0)
Grade 1	7 (41.2)	1 (20.0)
Grade 2	1 (5.9)	0
Median CRS duration, days (range)	2.5 (0–7)	1.0 (1–1)
CRS management	4 (23.5)	0
Tocilizumab	2 (11.8)	0
Steroids	1 (5.9)	0
Steroids + tocilizumab	1 (5.9)	0

CRS was graded using the ASTCT criteria [20]

*ASTCT* American Society for Transplantation and Cellular Therapy, *CRS* cytokine release syndrome

In the FLMOON-3 cohort, all safety-evaluable patients (n = 5) experienced an AE (Table 3). The most common AEs were ISRs, which occurred in all patients (100%), and all were Grade 1 in severity. No AEs leading to mosunetuzumab discontinuation occurred; one (20.0%) patient experienced an AE leading to dose modification/interruption (Table 3). Serious AEs were reported in one (20.0%) patient (viral pneumonia), and one (20.0%) patient experienced a Grade 3 AE (viral pneumonia); no Grade 4 AEs occurred (Table 4). No immune cell-associated neurotoxicity syndrome events occurred. One (20.0%) patient experienced a CRS event, which was Grade 1 in severity and occurred between Cycle 1 Day 8 and Day 14 (Table 5; Fig. 2b), this patient did not require treatment for the management of the CRS event (Table 5). No Grade 5 (fatal) AEs occurred.

### Pharmacokinetics

**Fig. 3:**
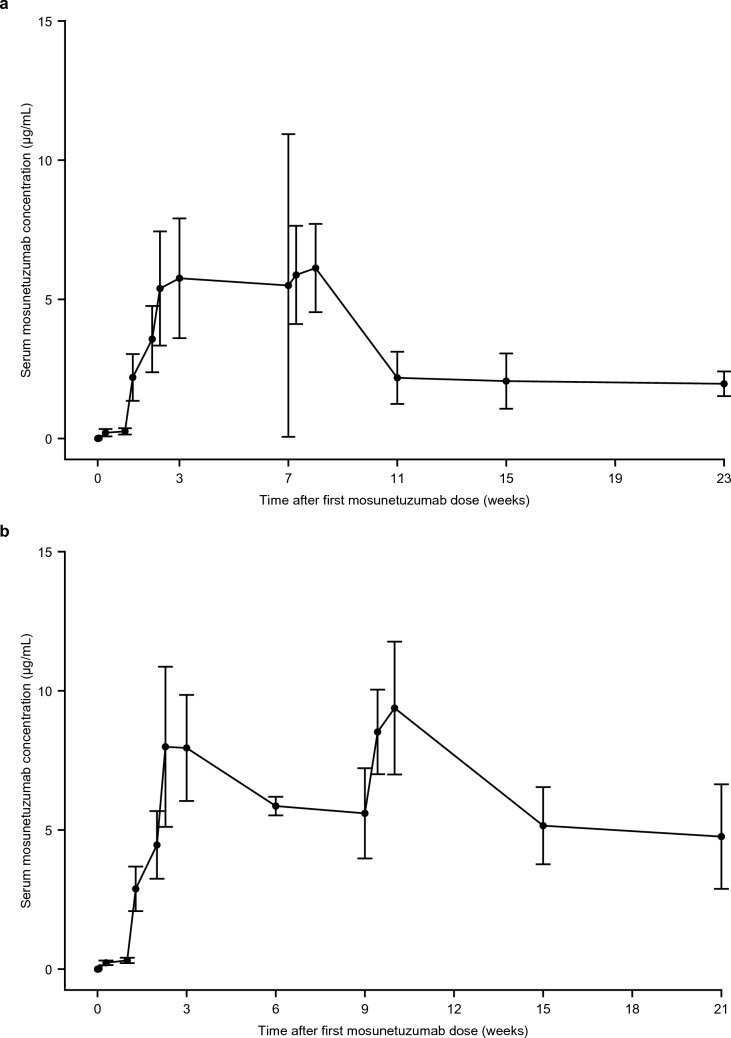
Time profiles of mean mosunetuzumab serum concentrations in **a** the FLMOON-2 (n = 17) and **b** the FLMOON-3 (n = 5) cohorts

In both cohorts, mosunetuzumab serum concentration peaked after the third dose in Cycle 1 (Fig. 3a, b) and then reached a steady state with repeated dosing.

In the FLMOON-2 cohort, in Cycle 1, the mean C_max_ (± standard deviation [SD]) was 5.90 (± 2.03) μg/mL and median T_max_ was 21.0 days. In Cycle 3, the mean C_max_ (± SD) was 8.08 (± 4.59) μg/mL and median T_max_ was 1.9 days post-dose. Mean trough serum concentrations (± SD) on Day 1 of Cycle 4 and Cycle 5 were 2.18 (± 0.937) μg/mL and 2.06 (± 0.992) μg/mL, respectively.

In the FLMOON-3 cohort, from Cycle 1 to Cycle 2, mean C_max_ (± SD) was 8.64 (± 2.30) μg/mL and median T_max_ was 18.5 days after the first dose. In Cycle 4, the mean C_max_ (± SD) was 9.60 (± 2.31) μg/mL and median T_max_ was 6.9 days post-dose. Mean trough serum concentrations (± SD) on Day 1 of Cycle 3 and Cycle 4 were 5.86 (± 0.334) μg/mL and 5.60 (± 1.62) μg/mL, respectively.

## Discussion

In this study (JO40295) of Japanese patients with R/R FL, mosunetuzumab demonstrated promising efficacy in combination with lenalidomide and as a monotherapy, with high response rates in both the FLMOON-2 and FLMOON-3 cohorts (CR rate: 92.3 and 100%, respectively), consistent with studies in global populations [8, 12, 14].

Mosunetuzumab IV previously demonstrated high ORR and CR rates of 80.0% (n = 72/90) and 60.0% (n = 54/90), respectively, in a global Phase I/II study (GO29781) of mosunetuzumab monotherapy in patients with R/R FL, after ≥ 2 prior therapies [8, 9]. Consistent with this, an ORR of 78.9% (n = 15/19) and CR rate of 68.4% (n = 13/19) were reported in Japanese patients with R/R FL after ≥ 2 prior lines of therapy, treated with mosunetuzumab IV monotherapy, from the FLMOON-1 cohort of the Phase I study (JO40295) [10]. In the pivotal cohort of GO29781, mosunetuzumab SC demonstrated an ORR of 74% and CR rate of 59% in 94 patients with R/R FL after ≥ 2 prior lines of therapy [14].

Mosunetuzumab in combination with lenalidomide is currently under investigation in patients with R/R FL after ≥ 1 prior line of therapy in both a Phase Ib/II study (CO41942) and the Phase III CELESTIMO study. Preliminary efficacy results from the Phase Ib/II study showed high response rates (ORR: 92%; CR rate: 77%), which were consistent with those seen in the FLMOON-2 cohort of the current study [12].

In this study, mosunetuzumab SC demonstrated a manageable safety profile, consistent with previous reports of mosunetuzumab in combination with lenalidomide and as a monotherapy, and no new safety signals were identified in either cohort [12, 14]. No AEs leading to study treatment discontinuation or fatal AEs occurred. In both cohorts, ISRs were the most common AEs and all were Grade 1 in severity. The incidence of CRS was similar to previous reports; events were predominantly Grade 1 and manageable [12, 14].

In a global Phase I/II study, mosunetuzumab SC demonstrated a non-inferior pharmacokinetic profile versus mosunetuzumab IV based on trough serum concentration in Cycle 3 and model-predicted cumulative area under the serum concentration–time curve from Cycle 1 to 4, in patients with R/R FL after ≥ 2 prior therapies [14]. In the current study, the pharmacokinetic data showed that serum concentrations in Cycle 1 peaked with the third dose of mosunetuzumab and then reached a steady state with repeated dosing. The mean C_max_ was decreased by more than 60% in the FLMOON-3 cohort (8.64 μg/mL) compared with IV administration (26.2 μg/mL) in patients from the FLMOON-1 study [10].

The main limitation of the current study was the small sample size in each cohort, with 17 safety-evaluable (n = 13 efficacy-evaluable) patients in the FLMOON-2 cohort and five safety-evaluable (n = 5 efficacy-evaluable) patients in the FLMOON-3 cohort. Another limitation was the short median follow-up in both cohorts (FLMOON-2: 5.9 months; FLMOON-3: 6.4 months), meaning data on response durability and important toxicities such as B-cell aplasia and hypogammaglobulinemia were unavailable at this analysis; further follow-up is therefore required. A strength of this study is the pre-planned inclusion of pharmacokinetic analyses of mosunetuzumab in combination with lenalidomide, and as a monotherapy. Also, published data on mosunetuzumab SC are limited and this paper is the first to evaluate mosunetuzumab SC in the Asia–Pacific region.

## Conclusion

Mosunetuzumab SC, in combination with lenalidomide and as a monotherapy, demonstrated high response rates in this analysis, comparable to those reported in previous studies of mosunetuzumab in global populations [12, 14]. The safety profile was consistent with previous reports, with CRS being a common but manageable AE and no new AEs of interest were observed. These promising results support the further development and potential use of mosunetuzumab SC in Japanese patients with R/R FL.

## Supplementary Information

Below is the link to the electronic supplementary material.

Supplementary file1 (DOCX 37 KB)

## Data Availability

Qualified researchers may request access to individual patient-level data through the clinical study data request platform (www.clinicalstudydatarequest.com). For further details on Chugai’s Data Sharing Policy and how to request access to related clinical study documents, see www.chugai-pharm.co.jp/english/profile/rd/ctds_request.html.
