# Supplementary material for: Efficacy and safety of subcutaneous mosunetuzumab in combination with lenalidomide and as a monotherapy in Japanese patients with relapsed/refractory follicular lymphoma
Source: Int J Clin Oncol. 2026 Jan 12;31(3):456–66. doi: 10.1007/s10147-025-02957-1 (PMC12932324; doi:10.1007/s10147-025-02957-1)
Supplement: Supplementary file 1 — Supplementary file1 (DOCX 37 KB) [file 10147_2025_2957_MOESM1_ESM.docx]

**Online Resource: Efficacy and safety of subcutaneous mosunetuzumab in combination with lenalidomide and as a monotherapy in Japanese patients with relapsed/refractory follicular lymphoma**

**Authors:** Shinichi Makita,^1^ Koji Izutsu,^1^ Yuko Mishima,^2^ Takahiro Kumode,^3^ Junya Kuroda,^4^ Nobuhiro Kanemura,^5^ Noriko Fukuhara,^6^ Kazuyuki Shimada,^7^ Chiemi Mori,^8^ Atsuko Kawasaki,^8^ Takeshi Miyake,^8^ Dai Maruyama^2^

**Affiliations:**

^1^Department of Hematology, National Cancer Center Hospital, Tokyo, Japan; ^2^Department of Hematology Oncology, Cancer Institute Hospital, Japanese Foundation for Cancer Research, Tokyo, Japan; ^3^Department of Hematology and Rheumatology, Faculty of Medicine, Kindai University, Osaka, Japan; ^4^Division of Hematology and Oncology, Department of Medicine, Kyoto Prefectural University of Medicine, Kyoto, Japan; ^5^Department of Hematology and Infectious Disease, Gifu University Hospital, Gifu, Japan; ^6^Department of Hematology, Tohoku University, Sendai, Japan; ^7^Department of Hematology and Oncology, Nagoya University Graduate School of Medicine, Nagoya, Japan; ^8^Chugai Pharmaceutical Co., Ltd, Tokyo, Japan.

**Corresponding author:** Shinichi Makita

Department of Hematology, National Cancer Center Hospital, Tokyo, Japan

**Email:** [smakita@ncc.go.jp](mailto:smakita@ncc.go.jp)

**Journal:** *International Journal of Clinical Oncology*

**Inclusion Criteria**

Patients who met all of the following criteria were eligible for inclusion in the study:

1. Have received a full explanation of the details of the study and given their written consent
2. Aged ≥18 years at the time of informed consent
3. Eastern Cooperative Oncology Group performance status of 0, 1 or 2
4. Life expectancy of at least 12 weeks after the day of enrollment
5. Histologically confirmed CD20 positive, Grade 1–3a, relapsed/refractory follicular lymphoma
6. FLMOON-3 cohort: Prior systemic lymphoma therapy with at least two regimens including an anti-CD20 targeted therapy and an alkylating agent
7. FLMOON-2 cohort: Prior systemic lymphoma therapy with at least one regimen including immunotherapy or chemoimmunotherapy. Treatment for systemic lymphoma refers to rituximab monotherapy, chemotherapy with or without rituximab, and radioimmunotherapy with ibritumomab tiuxetan, etc. Treatment of systemic lymphoma does not include local radiotherapy for limited stage disease or treatment such as antibiotics for pathogen eradication
8. FLMOON-2 cohort: Judged to require systemic therapy based on the evaluation by the investigator or sub-investigator, according to tumor size and/or, Groupe d'Etude des Lymphomes Folliculaires (GELF) criteria
9. Able to provide tumor tissue samples that meet all of the following criteria:

- Formalin-fixed, paraffin-embedded blocks of tumor tissue samples or three unstained slides
- Samples collected after the last dose of prior treatment (however, if this procedure cannot be performed, a sample collected before the last dose of the previous treatment is acceptable)
- No evidence of transformation or change in histological type from the time the tumor tissue sample was obtained

1. At least one measurable lesion (measurable in two perpendicular directions by computed tomography [CT] or magnetic resonance imaging [MRI] and >1.5cm in the longest diameter for a lymph node lesion or >1.0cm for an extranodal lesion)
2. Vital organ function meeting all of the following criteria below (except for findings as judged by the investigator or sub-investigator to the underlying disease or other diseases (e.g. immune thrombocytopenia and/or bone marrow involvement). If more than one test result is available during this period, the latest one before enrollment will be used

- Neutrophil count: ≥1,000/μL
- Platelet count: ≥75,000/μL
- Hemoglobin: 9.0g/dL
- Aspartate transaminase and alanine transaminase: ≤3 times the study site upper limit of normal
- Total bilirubin: ≤1.5 times the study site upper limit of the site reference range; patients with a history of Gilbert’s syndrome are allowed to enroll if elevated total bilirubin is accompanied by elevated indirect bilirubin
- Serum creatinine: ≤upper limit of the study site reference value or creatinine clearance 40mL/min. Measured values or creatinine clearance calculated using the Cockcroft-Gault formula:
- Male: [(140 − age) × body weight (kg) / [72 × serum creatinine (mg/dL)]
- Female: [(140 − age) × body weight (kg) x 0.85 / [72 × serum creatinine (mg/dL)]

1. By the scheduled Cycle 1 Day 1, at least one of the following periods of time must have passed since the end date of the prior treatment or procedure

- Surgical procedures: 4 weeks. This includes major surgeries such as thoracotomy, laparotomy or laparoscopic organ resection. However, if a protocol-specified biopsy (tumor biopsy or bone marrow biopsy) has been performed during this period, the patient may be enrolled if the event has resolved to the point where the investigator or sub-investigator judges that there is no bleeding risk at the time of enrollment
- Chemotherapy and other anti-cancer therapies: five times the drug’s half-life or 4 weeks, whichever is shorter (including molecularly targeted drugs)
- Antibody therapy for the treatment of malignancies: 4 weeks (including monoclonal antibodies, radioimmunoconjugates or antibody-drug conjugates). Excluding immuno-oncology therapies specified below
  - Cancer immunotherapy: five times the drug’s half-life or 12 weeks, whichever is shorter (anti-PD-1/PD-L1 antibody drugs, anti-CTLA-4 antibody drugs, etc.)
  - Co-stimulatory agonists (anti-CD137 antibody drugs, anti-CD27 antibody drugs, anti-GITR antibody drugs or anti-CD40 antibody drugs, etc.) and all other immuno-oncology therapies (including immunomodulators such as IFN-α, IFN-γ, IL-2, BRM therapy, cancer vaccine therapy, etc.)
- Radiotherapy: 2 weeks
  - If there is only one measurable lesion and no unequivocal progression in the irradiated lesion: 4 weeks
- Autologous stem cell transplant: 12 weeks
- Platelet transfusion: 2 weeks
- Red blood cell transfusion: 3 weeks
- Immunosuppressive therapy: 2 weeks
  - Including prednisolone, cyclophosphamide, azathioprine, methotrexate, thalidomide, anti-TNF-α agents including the drug product. However, continued use of the following immunosuppressive therapies is allowed:
- Inhaled corticosteroids
- 10mg/days or less of prednisolone or equivalent
- Systemic immunosuppressants for acute disease (nausea, B symptoms, etc.)
- Mineralocorticoids for the management of orthostatic hypotension
- Corticosteroids for management of adrenal insufficiency
- Live vaccines, attenuated vaccines, inactivated vaccines, toxoids: 4 weeks
- Alemtuzumab, fludarabine, cladribine, and pentostatin: 24 weeks, unless fludarabine was used as lymphodepleting therapy prior to chimeric antigen receptor (CAR) T-cell therapy
- Other investigational products: five times the drug half-life or 4 weeks, whichever is shorter
- CAR T-cell therapy: 30 days
- FLMOON-2 cohort: Lenalidomide: 12 months

**Exclusion Criteria**

Patients who met any of the following criteria were excluded from study entry:

1. History of hypersensitivity to the excipients of mosunetuzumab, including histidine acetate, sucrose, polysorbate and L-methionine
2. Known history of severe allergic or anaphylactic reactions to monoclonal antibody therapy (chimeric antibodies, humanized antibodies and human antibodies) or fusion proteins
3. FLMOON-2 cohort: Risk of hypersensitivity to lenalidomide and excipients of lenalidomide
4. FLMOON-2 cohort: Documented resistance to lenalidomide (non-response) without partial or complete response, or relapse within 6 months after treatment
5. History or presence of central nervous system (CNS) lymphoma
6. History of a malignancy that could affect compliance with the protocol or interpretation of results, with the exception of the following malignancies if previously treated with curative intent:

- A history of curatively treated basal or squamous cell carcinoma of the skin, carcinoma in situ of the cervix, or ductal carcinoma in situ of the breast with favorable prognosis
- Stage I malignant melanoma, low grade early stage localized prostate cancer
- Any other previously treated malignancy that has been in remission without therapy for at least 2 years prior to the first dose of mosunetuzumab

1. Persistent treatment-emergent adverse reactions associated with prior treatment that are rated Grade ≥2 as according to the National Cancer Institute Common Terminology Criteria for Adverse Events (NCI CTCAE) Version 4.03. However, patients with alopecia and lymphocyte count decrease are permitted
2. History of the following immune-mediated adverse events related to prior immunotherapy treatment:

- Events of Grade ≥3 according to NCI CTCAE v4.03 (excluding endocrinopathies manageable with hormone replacement therapy)
- Grade 1–2 according to NCI CTCAE v4.03 that did not resolve following discontinuation of immunotherapy

1. Active infections requiring systemic treatment with antimicrobials, antifungals, antivirals, etc., or history of these diseases within 4 weeks before enrollment. Patients with superficial fungal infections are permitted. Confirmation of infection via SARS-CoV-2 PCR or antigen test is recommended.
2. Presence or history of autoimmune disease; however, the following are permitted:

- Patients with a history of autoimmune disease or well controlled autoimmune disease, with at least 12 months since the last immunosuppressive therapy may be eligible if deemed safe by the investigator
- History of hypothyroidism controlled with stable doses of thyroid hormone replacement
- History of disease-related immune thrombocytopenic purpura or autoimmune hemolytic anemia
- Skin rash that covers ≤10% of the body surface area (remains in skin lesions, such as eczema, psoriasis, chronic uncomplicated lichenoid, vitiligo, etc.), controlled with low-potency topical steroid, with no acute exacerbation within 12 months of enrollment, and without the need for psoralen, ultraviolet light A irradiation, methotrexate, retinoids, biologics, oral calcineurin inhibitors, or high potency steroids

1. Current active tuberculosis
2. Prior allogeneic hematopoietic stem cell transplantation or organ transplantation
3. Positive test results for human immunodeficiency virus antibodies
4. Positive test results for hepatitis B surface (HBs) antigen, HBs antibodies or hepatitis B core HBc antibodies. However, patients with positive HBs antibodies that are clearly due to vaccination, or patients with positive HBs antibodies and/or HBc antibodies only and undetectable hepatitis B virus-DNA are eligible
5. Positive test results for hepatitis C virus (HCV) antibodies. However, patients who are positive for HCV antibodies and negative for HCV-RNA can be enrolled
6. Evident or suspected chronic active Epstein-Barr virus infection
7. A confirmed diagnosis of progressive multifocal leukoencephalopathy
8. History or presence of hemophagocytic lymphohistiocytosis or macrophage activation syndrome
9. Presence of significant, uncontrolled pulmonary disease that could confound interpretation of study results, such as current or history of obstructive pulmonary disease or lung disease requiring treatment such as symptomatic bronchospasm
10. Current cardiac diseases, arrhythmias, or unstable angina of New York Heart Association Class III or higher, or history of myocardial infarction within 6 months prior to enrollment
11. Current or previous CNS disease (stroke, epilepsy, CNS vasculitis, neurodegenerative disease, etc.)

- Patients with a history of stroke who have not experienced a stroke or transient ischemic attack in the past 2 years, and with no residual neurological deficit in the opinion of the investigator or sub-investigator, may be enrolled
- Patients with a history of epilepsy who have not had seizures in the past year while not being treated with antiepileptic drugs may be enrolled

1. FLMOON-3 cohort: Pregnant or lactating women (except for lactating women who have consented to stop breastfeeding from the start of the study intervention until 90 days after mosunetuzumab, or the last dose of tocilizumab, whichever is longer
2. FLMOON-2 cohort: Pregnant or lactating women
3. FLMOON-3 cohort: Women with a positive pregnancy test (women who have not reached postmenopausal status [amenorrhea for ≥12 months without an identified alternative cause] and who have not undergone an oophorectomy and/or hysterectomy will be considered women of childbearing potential and will undergo pregnancy testing. Women who experienced amenorrhea in the last 12 months before enrollment but could still be pregnant because the amenorrhea was chemically induced or due to other relevant reasons must also take a pregnancy test or if the pregnancy test is negative, it is judged possible to be pregnant based on the interview, etc. by the investigator/sub-investigator)
4. FLMOON-2 cohort: Women with a positive pregnancy test (women who have not reached postmenopausal status [amenorrhea for at least 24 months without an identified alternative cause] and who have not undergone an oophorectomy and/or hysterectomy will be considered women of childbearing potential and will undergo pregnancy testing. Women who experienced amenorrhea in the last 24 months before enrollment but could still be pregnant because the amenorrhea was chemically induced or due to other relevant reasons must also take a pregnancy test or if the pregnancy test is negative, it is judged possible to be pregnant based on the interview, etc. by the investigator/sub-investigator)
5. FLMOON-3 cohort: Women of childbearing potential who are not willing to abstain from heterosexual intercourse or to use adequate contraceptive methods with a failure rate of <1% per year, or unwilling not to donate eggs for the duration of the study and for 90 days after the last dose of mosunetuzumab or tocilizumab, whichever is longer
6. FLMOON-2 cohort: Women of childbearing potential who are not willing to abstain from heterosexual intercourse or to use adequate contraceptive methods with a failure rate of <1% per year, or unwilling not to donate eggs from 28 days before Cycle 1 Day 1, throughout the study, and for 90 days after the last dose of mosunetuzumab or tocilizumab, or 28 days after the last dose of lenalidomide, whichever is longer
7. FLMOON-3 cohort: Men who are not willing to abstain from heterosexual intercourse or to use an adequate contraceptive method with a failure rate of <1% per year, or unwilling not to donate sperm during the study and for 60 days after the last dose of mosunetuzumab or 90 days after the last dose of tocilizumab, whichever is longer
8. FLMOON-2 cohort: Men who are not willing to abstain from heterosexual intercourse or to use an adequate contraceptive method or not willing to donate sperm during the study and for 60 days after the last dose of mosunetuzumab or 90 days after the last dose of tocilizumab or 28 days after the last dose of lenalidomide, whichever is longer
9. Prior treatment with a bispecific anti-CD20 antibody targeting both CD20 and CD3
10. Any patient judged by the investigator or sub-investigator to be ineligible for participation in this study for any other reason
